# PKGPT: Expert-Orchestrated Recursive LLM Agent for Automated NONMEM PopPK Modeling with Human Benchmarking

**DOI:** 10.3390/pharmaceutics18040501

**Published:** 2026-04-18

**Authors:** Hoyoung Kwack, Hyunseung Kong, Jiwoo Lim, Byoung-Tak Zhang, Jongsung Hahn, Min Jung Chang

**Affiliations:** 1 Department of Pharmacy and Yonsei Institute of Pharmaceutical Sciences, Yonsei University, Incheon 21983, Republic of Korea; hoyoung0104@yonsei.ac.kr; 2 Interdisciplinary Program in Bioinformatics, Seoul National University, Seoul 08826, Republic of Korea; hskong@snu.ac.kr (H.K.); btzhang@bi.snu.ac.kr (B.-T.Z.); 3 Department of Integrative Biotechnology, College of Pharmacy, Yonsei University, Incheon 21983, Republic of Korea; lmjw@yonsei.ac.kr; 4 Department of Computer Science, Seoul National University, Seoul 08826, Republic of Korea; 5 College of Pharmacy, Jeonbuk National University, Jeonju 54896, Republic of Korea; 6 Graduate Program of Industrial Pharmaceutical Science, Yonsei University, Incheon 21983, Republic of Korea; 7 Department of Pharmaceutical Medicine and Regulatory Science, Yonsei University, Incheon 21983, Republic of Korea

**Keywords:** population pharmacokinetics, NONMEM, large language model, agentic AI, model automation, recursive self-correction, human-in-the-loop, model informed drug development

## Abstract

**Background/Objectives:** Population pharmacokinetic (PopPK) modeling in NONMEM requires iterative, expertise-dependent workflows. Naïve zero-shot prompting of general-purpose large language models (LLMs) typically produces NONMEM code that fails to execute. This study introduces PKGPT, a recursive agentic LLM system designed to automate NONMEM-based PopPK model development and benchmarks its performance against human expert models. **Methods:** PKGPT, powered by Google’s Gemini 3.0 Flash, embeds pharmacometrics expertise into phase-specific expert-agent prompts orchestrated across five sequential phases: base model establishment, structural diagnostics, overfitting reduction, random-effects optimization, and covariate analysis. The system recursively executes NONMEM, parses outputs, and iteratively refines control streams. PKGPT was evaluated on three public datasets (warfarin, theophylline, and tobramycin) and benchmarked against independently developed human expert models. **Results:** PKGPT consistently produced executable, converging NONMEM models across all three datasets. In warfarin, both PKGPT and the human expert selected a one-compartment oral structure (ADVAN2), but the expert achieved a lower OFV (294.41 vs. 484.43) via covariate scaling. In theophylline, PKGPT produced parameter estimates close to the expert solution (Ka = 1.59 vs. 1.46 h^−1^; CL = 0.0399 vs. 0.0404 L/h/kg). In tobramycin, PKGPT correctly identified a two-compartment structure but produced physiologically implausible peripheral volume estimates (V2 = 149 L vs. expert’s 13.2 L). Across datasets, PKGPT did not identify clinically established covariates, and run-to-run reproducibility was variable. **Conclusions:** PKGPT substantially improves the robustness and usability of LLM-generated NONMEM code compared with naïve zero-shot prompting, accelerating model drafting and iterative refinement, but physiological plausibility and clinical interpretability still require a human-in-the-loop oversight.

## 1. Introduction

Population pharmacokinetic (PopPK) modeling is a standard component of model-informed drug development (MIDD) and regulatory decision-making, enabling characterization of between-subject variability, identification of clinically relevant covariates, and development of dosing recommendations across diverse patient populations [1,2,3,4,5,6,7,8]. Both the FDA and EMA have issued guidance emphasizing the role of PopPK analyses in supporting labeling claims and dose adjustments [7,8], and these analyses are now embedded throughout drug development programs [9,10,11].

Despite its established value, PopPK model development remains a labor-intensive and expertise-dependent process. The workflow is inherently iterative, requiring modelers to cycle through data interpretation, structural model specification, random-effects modeling, covariate selection, and diagnostic evaluation, often with multiple rounds of refinement before arriving at a final model [12,13]. This process is further complicated by the need to balance statistical fit against physiological plausibility, ensure model stability across estimation methods, and identify clinically meaningful covariate relationships from among numerous candidate predictors [14]. Surveys of industry practitioners indicate that covariate modeling alone—a single component of the overall workflow—can consume substantial time and resources, with the stepwise covariate model (SCM) building procedure remaining the most common approach despite its computational demands and susceptibility to local optima [15,16].

To reduce this burden, the pharmacometrics community has developed an extensive ecosystem of auxiliary tools. Workbenches such as Perl-speaks-NONMEM (PsN), Pirana, and Xpose provide programmatic interfaces for batch execution, automated covariate search procedures, diagnostic visualization, and output parsing [17]. While these tools enhance workflow efficiency, they still require a pre-constructed, syntactically correct control stream, a bottleneck that large language models (LLMs) are uniquely positioned to address. LLMs have demonstrated remarkable capabilities in code generation and program synthesis across a wide range of programming tasks [18]. More recently, “agentic” LLM systems that combine task decomposition, tool use, and feedback-driven refinement have shown particular promise for complex software engineering tasks [19,20]. Frameworks that interleave reasoning with actions and external feedback can improve reliability by enabling iterative self-correction when execution outcomes can be observed and incorporated into subsequent revisions [21,22]. Agent–computer interfaces that provide structured feedback and constrained action spaces have proven essential for enabling LLMs to navigate real-world codebases and debug errors autonomously [23].

Motivated by these developments, this study introduces PKGPT, an agentic LLM system designed to produce executable NONMEM PopPK models and refine them through recursive, execution-in-the-loop iteration. Unlike generic LLM prompting approaches that treat NONMEM coding as a single-pass text generation task, PKGPT explicitly encodes pharmacometrics expertise into multiple specialized phase-specific expert-agent prompts and coordinates them across the full modeling lifecycle. The system transforms NONMEM error messages and run outputs into targeted repairs and structured model improvements, implementing phase-specific guidance that mirrors the sequential decision-making workflow of an experienced pharmacometrician. Critically, PKGPT addresses the fundamental challenge that has limited LLM utility in pharmacometrics: bridging the gap between the hallucinated syntax commonly produced by general-purpose LLMs and the rigid NM-TRAN-specific constraints required for successful parameter estimation. We evaluate PKGPT on three public PopPK datasets and benchmark its best models against independently developed human expert models, focusing on executability, iterative convergence, and the trade-off between objective function improvements and physiological plausibility.

## 2. Methods

PKGPT is an automated PopPK modeling system that generates, executes, and iteratively refines NONMEM control streams through a recursive execution-in-the-loop optimization process. The core design principle is to replace “zero-shot” LLM code generation with an orchestrated set of specialized, phase-specific expert prompts that emulate the sequential decision-making workflow of a PopPK modeler. The overall loop follows a fixed sequence of steps—NONMEM execution, output parsing, issue diagnosis, targeted model revision, and convergence evaluation—repeated until convergence criteria are met or a maximum iteration budget is exhausted.

### 2.1. Datasets and Formatting

We evaluated PKGPT using three standard PK datasets distributed by the Monolix Suite 2024R1 (Lixoft SAS, Antony, France) dataset repository: warfarin, theophylline, and tobramycin (dataset URLs provided in the Supplementary Material). All datasets obtained from the Monolix Suite repository were converted to NONMEM-style tabular format (CSV files), with required columns including ID, TIME, DV, AMT, EVID, and MDV, and optional columns such as CMT, RATE, and subject-level covariates (e.g., WT, AGE, SEX).

### 2.2. Data Introspection and Structural Inference

Before any control stream is generated, PKGPT performs dataset introspection to identify NONMEM-standard columns, infer covariates, and compute summary statistics. A key component is the automatic assessment of PK profile complexity to recommend an initial compartment model. Because PKGPT must select an initial structural model before any NONMEM execution, the standard approach of comparing OFV between fitted one- and two-compartment models is not available at this stage. Instead, PKGPT employs a pre-fitting heuristic based on the observed concentration-time profiles: Specifically, PKGPT estimates a terminal elimination slope (λ_z_) via log-linear regression on tail concentrations and an early distribution slope (λ_early_) after C_max_, then uses BIC-based comparison and conservative thresholds to recommend a 1-compartment versus 2-compartment structure. The default recommendation is upgraded to 2-compartment only when ΔBIC > 20, λ_early_/λ_z_ ≥ 3.0, and R^2^ ≥ 0.98. These thresholds were empirically calibrated to prioritize specificity over sensitivity in compartment upgrades and are configurable parameters within the system. All three criteria must be simultaneously satisfied (AND logic), implementing a high-specificity decision rule that minimizes premature structural escalation in automated pipelines where human oversight is limited. Specifically, the ΔBIC > 20 criterion substantially exceeds the conventional ΔBIC > 10 threshold for “very strong” evidence on the Kass and Raftery (1995) scale, where values exceeding 10 constitute “decisive” evidence; doubling this threshold reflects deliberate conservatism appropriate for automated pipelines where false-positive compartment upgrades could propagate through subsequent phases without human oversight [24], reflecting a deliberate bias toward parsimonious models that avoids premature structural escalation in automated pipelines where human oversight is limited. The slope ratio (λ_early_/λ_z_ ≥ 3.0) ensures that the two-compartment recommendation requires clear pharmacokinetic evidence of a distinct distribution phase, and the R^2^ ≥ 0.98 requirement confirms adequate fit of the terminal log-linear regression. This initial recommendation serves as a starting point and is subsequently re-evaluated in Phase 2, where residual diagnostics from the fitted model can trigger compartment upgrades (e.g., ADVAN2 → ADVAN4) if structural misspecification is detected.

### 2.3. LLM Backend and Expert-Agent Orchestration

PKGPT interfaces with an LLM through an API client that supports multiple model configurations selectable via a command-line parameter (e.g., flash, flash-lite, pro). In this study, we used Google’s Gemini 3.0 Flash (Google LLC, Mountain View, CA, USA) model as the underlying engine, selected for its optimal trade-off between reasoning capability, inference latency, and cost-efficiency during iterative refinement tasks. The system also includes robustness features for API rate limits, including automatic retries with exponential backoff. This choice reflects the practical demands of iterative refinement, where each modeling run involves 20–50 API calls for code generation, output parsing, and diagnostic interpretation, making inference cost and latency critical considerations. Selection was guided by the LiveBench benchmark (https://livebench.ai/#/?highunseenbias=true, accessed on 15 February 2025), which ranked Gemini 3.0 Flash’s coding and instruction-following performance within the tier of frontier models while offering substantially lower per-call cost compared to premium alternatives such as GPT-4o or the Claude 3.5 series. Importantly, the primary objective of this study was to evaluate the feasibility of pharmacometric model automation through an agentic LLM architecture—not to benchmark LLM performance *per se*. Accordingly, the choice of a specific LLM backend was treated as an implementation decision rather than an experimental variable, and the system was deliberately designed so that the phase-based orchestration framework and execution-in-the-loop logic are model-agnostic, allowing straightforward substitution of the underlying LLM without modifying the pharmacometric workflow. We acknowledge that more capable frontier models (e.g., GPT-5, Claude 4 series) may yield improvements in specific sub-tasks—particularly covariate identification, where domain-specific pharmacological reasoning beyond syntactic code generation is required, and in run-to-run structural consistency, where stronger instruction-following may reduce Phase 1 initialization variance. Whether such improvements would meaningfully close the performance gap with human experts on physiological plausibility and clinical covariate detection is an empirical question that systematic cross-model benchmarking, planned as a direct follow-up to this work, will address.

Rather than relying on a single generic prompt, PKGPT encodes domain knowledge and structured troubleshooting heuristics into phase-specific guidance that functions as a collection of specialized “expert agents.” These agents are not separate models; instead, they are distinct prompt modules that are invoked conditionally based on the current modeling phase and the most recent NONMEM diagnostics. The guidance explicitly targets common NONMEM failure modes (syntax issues, boundary problems, estimation instability), structural misspecification patterns (e.g., U-shaped residuals, multiphasic decline), overfitting signatures (e.g., extreme shrinkage, collapsed OMEGAs, markedly reduced OFV), IIV structure refinement, and covariate testing logic.

### 2.4. Recursive Optimization Loop

The fundamental engine of PKGPT is a recursive loop that automates the cycle of model generation, execution, and diagnostic feedback, mirroring the iterative review process that pharmacometricians perform manually after each NONMEM run. At each iteration, PKGPT executes NONMEM via a configurable nmfe command (default nmfe75) and monitors output generation for completeness using file stability and the presence of NONMEM termination markers, such as the “Stop Time” stamp. Following execution, the system employs AI-assisted parsing with a robust regex fallback to extract the Objective Function Value (OFV), minimization status, parameter estimates, RSE%, ETA shrinkage, and error signatures. These parsing targets correspond to the standard diagnostic checklist that pharmacometricians routinely evaluate after each NONMEM run [14], ensuring that the automated assessment aligns with established model evaluation practice.

To ensure systematic improvement, PKGPT maintains a comprehensive iteration history and selects the best model based on a composite quality score that prioritizes stable minimization and the avoidance of numerical pathologies. Overfitting safeguards are explicitly encoded into the scoring logic. Physiologically implausible objective function values (e.g., ΔOFV < −50 in small datasets) are penalized, as extremely reduced OFV in limited sample sizes may indicate that the random-effects structure is fitting residual noise rather than true interindividual variability. Pharmpy AMD—autometic model development tool—similarly flags models exceeding condition number thresholds of 1000 as a quality concern [25]. Phase-specific overfitting criteria, including ETA shrinkage thresholds, are described in Section 2.6.

The recursive loop terminates when technical criteria are satisfied: minimization is successful with no major warnings, and iteration-to-iteration OFV change falls below a threshold (ΔOFV < 0.1), indicating diminishing returns from further modifications, parameter bounds are satisfied, and a minimum iteration count has been completed. This external stopping criterion operates at the level of the LLM’s iterative loop—where each iteration represents a complete NONMEM run with a modified control stream—and is conceptually analogous to convergence mechanisms at other levels: NONMEM’s NSIG parameter terminates estimation when objective function change falls below a specified number of significant digits [26], SAMBA terminates when no improvement in information criterion is achieved [27], and pyDarwin concludes after successive generations without fitness improvement [28]. A recent machine-learning-based approach [29] adopted a comparable two-level strategy, applying NSIG = 3 for internal NONMEM convergence while relying on pyDarwin’s algorithm-level stopping rules for the external search.

### 2.5. AI-Based Model Quality Evaluation

At each iteration, PKGPT performs AI-based model quality evaluation using a composite score (0–100) across five dimensions: Convergence (30% weight), assessing minimization success and covariance step completion; Shrinkage (25% weight), evaluating ETA shrinkage relative to dataset size with context-dependent thresholds; Stability (20% weight), detecting overfitting signals such as implausibly reduced OFV (ΔOFV < −50) or collapsed parameters; Precision (15% weight), examining parameter standard errors; and Utility (10% weight), evaluating model complexity appropriateness for the dataset size. Models are graded on a scale from A (90–100, excellent) through F (0–44, failed). The optimization continues if the model exhibits critical failures (minimization failed, shrinkage >90% with multiple OMEGA terms) or poor quality for dataset size and stops when quality score ≥65 with successful minimization and no critical overfitting signs. The relative ordering of these dimensions reflects the established hierarchy of model evaluation criteria in pharmacometric practice, where successful minimization is a prerequisite for all subsequent diagnostics [14]. Shrinkage (25%) is calibrated against the 20–30% EBE-based diagnostic reliability threshold established by Savic and Karlsson [30]. Stability and Precision thresholds are informed by the diagnostic framework of Karlsson and Savic [31]. Both the FDA [7] and EMA [8] guidelines emphasize convergence and parameter precision as fundamental evaluation elements, though neither prescribes specific numerical cutoffs. The priority weights were empirically calibrated based on this hierarchy, informed by the evaluation frameworks of existing automated tools: pyDarwin assigns binary penalties for convergence failure and condition number > 1000 [28], and a recent machine-learning-based approach [29] designed a composite penalty incorporating RSE, variability, and shrinkage to discourage implausible parameter values. As with pyDarwin’s user-specified penalties [28], these default weights and thresholds are configurable, allowing adaptation to different dataset characteristics or modeling objectives.

### 2.6. Phase-Based Orchestration and Forward-Only State Progression

The system coordinates model development through an explicit phase progression that emulates a manual PopPK workflow: (Phase 1) establishing a runnable base model, (Phase 2) diagnosing structural adequacy, (Phase 3) reducing overfitting if detected, (Phase 4) optimizing random effects/IIV structure, and (Phase 5) performing covariate analysis when prerequisites are satisfied. A key guardrail is that phases are constrained to move forward only (never backward), to prevent oscillatory loops in which later-phase refinements trigger repeated regressions to early-phase fixes. Phase transitions are driven by diagnostic criteria, including minimization success, ETA shrinkage thresholds, and OFV stability. Phase 3 (overfitting reduction) is entered when either of two conditions is met after Phases 1–2: shrinkage exceeds 95% or OFV falls below −50, both indicating potential overparameterization. Within Phase 3, model simplification strategies—including OMEGA parameter removal, error model reduction, and random-effect restructuring—are iteratively applied. Phase 3 is exited when shrinkage is reduced to <90% or OFV returns above −50, confirming that the overfitting signal has been resolved. The 95% entry threshold was chosen to target structural collapse, where OMEGA effectively approaches zero, rather than the 20–30% level at which Savic et al. [30] established that EBE-based diagnostics become unreliable. Applying this lower bound as a hard trigger would cause frequent, premature phase transitions—particularly for intermediate models where moderate shrinkage (30–70%) may resolve through subsequent LLM-guided refinements within the same phase. Specific examples of phase transition logic are illustrated in Section 3.

### 2.7. Covariate Modeling Procedure

Covariate analysis is gated by prerequisites, requiring a successful and stable base model with informative ETAs. When initiated, PKGPT performs stepwise covariate modeling using forward selection (ΔOFV > 3.84 for df = 1) and backward elimination (ΔOFV > 6.63 for df = 1) to maintain a balance between sensitivity and parsimony.

### 2.8. Implementation, Configuration, and Outputs

PKGPT is implemented in Python (3.12) and is designed to run with NONMEM 7.5 (ICON plc, Gaithersburg, MD, USA) for full execution. Each run produces iteration-stamped control streams and output files (e.g., _iterk.txt, _iterk.lst), ensuring a full audit trail of the optimization process, with the final best-performing model preserved as _final.txt.

### 2.9. Evaluation Framework: Benchmarking Against Human Expert Models

To evaluate the quality of PKGPT-generated models, we benchmarked them against independently developed human expert models for each of the three datasets. Both the human expert and PKGPT models were developed using the same software environment (NONMEM 7.5, executed via nmfe75) and the same NONMEM-formatted datasets described in Section 2.1, ensuring a comparable computational platform. The human expert modeler followed a conventional pharmacometrics workflow consisting of exploratory data analysis, physiologically informed structural model selection, sequential random-effects specification, and hypothesis-driven covariate screening based on established pharmacological knowledge. The expert models were developed independently and without access to any PKGPT outputs; conversely, PKGPT operated without knowledge of the expert solutions, ensuring an unbiased comparison.

To characterize the stochastic variability inherent in LLM-driven optimization, PKGPT was executed three times independently for each dataset, with each run initiated from a new LLM session to ensure independent initial conditions. This design reflects the stochastic nature of autoregressive text generation, which can produce different initial model specifications and optimization trajectories across runs. The best-performing run was selected based on the composite quality score described in Section 2.5, and this model was used for the primary comparison against the human expert model. Results from all three runs are reported in the Supplementary Tables (Tables S1–S3) to characterize run-to-run reproducibility and structural consistency.

## 3. Results

### 3.1. System Architecture and Workflow

**Figure 1:**
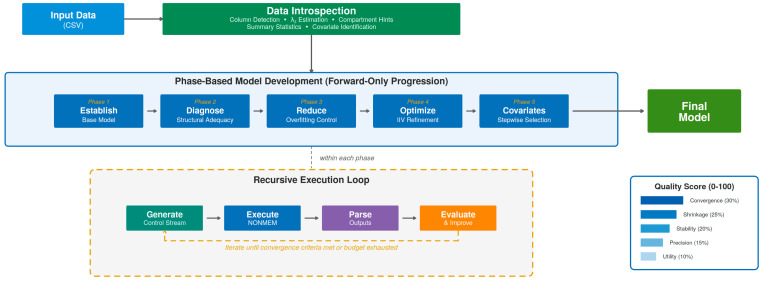
PKGPT system architecture and workflow. The recursive optimization loop consists of five phases: base model establishment, structural diagnostics, overfitting reduction, IIV optimization, and covariate analysis. The composite quality score (0–100) evaluates each model across five weighted dimensions: Convergence, Shrinkage, Stability, Precision, and Utility.

Figure 1 illustrates the PKGPT system architecture and its recursive optimization workflow. The framework implements an “execution-in-the-loop” design, where the LLM agent directly executes NONMEM, parses the resulting output files (.lst), and uses the extracted information—including OFV, parameter estimates, ETA shrinkage values, warnings, and error messages—to guide subsequent model modifications. This closed-loop execution–feedback–repair cycle enables automated error detection and iterative code refinement, substantially reducing manual intervention during the optimization process.

The framework implements a recursive, execution-in-the-loop architecture for automated PopPK modeling. Input data (CSV) first passes through a data introspection module that performs column detection, λz estimation via log-linear regression, and compartment hints based on BIC comparison to guide initial ADVAN selection. The phase-based model development follows a forward-only progression through five sequential phases: Establish (base model generation), Diagnose (structural adequacy), Reduce (overfitting control), Optimize (IIV refinement), and Covariates (stepwise covariate modeling). Within each phase, a recursive execution loop iterates through control stream generation, NONMEM execution, output parsing, and model evaluation until convergence criteria are met, progressively converging toward the final model. Model quality is assessed using a composite score (0–100) across five weighted dimensions: Convergence (30%), Shrinkage (25%), Stability (20%), Precision (15%), and Utility (10%).

Bar plots compare objective function values (OFV) achieved by human expert models (blue) and PKGPT (orange). For Warfarin, the human expert achieved a lower OFV (294.4 vs. 484.4) through allometric scaling and lag time. For Theophylline, PKGPT showed a modestly higher OFV (166.3 vs. 133.1) with comparable parameter estimates. For Tobramycin, PKGPT achieved a lower OFV (161.7 vs. 173.1) but with physiologically implausible peripheral volume estimates.

### 3.2. Phase Progression and Model Refinement

PKGPT orchestrates the model development process through five sequential phases, each dedicated to specific refinement objectives. In Phase 1 (Establish Base Model), the system performs data introspection, estimates terminal elimination slope (λ_z_) via log-linear regression, and applies a BIC-based comparison for initial compartment selection. It then generates an initial control stream with an appropriate ADVAN/TRANS specification. Phase 2 (Diagnose Structural Adequacy) evaluates residual patterns to detect structural misspecification. The detection of U-shaped residuals or evidence of biphasic decline triggers automated compartment upgrades (e.g., ADVAN2 → ADVAN4). Phase 3 (Reduce Overfitting) monitors overfitting indicators, including extreme ETA shrinkage (>90%), collapsed OMEGA terms, and physiologically implausible OFVs, applying model simplification when necessary. Phase 4 (Optimize IIV Structure) refines the random effects structure using dataset-size-dependent criteria—prioritizing diagonal Ω for small samples and block structures only when statistically warranted—combined with shrinkage-based retention thresholds. In Phase 5 (Covariate Analysis), upon satisfying stability prerequisites, the system performs stepwise covariate modeling using standard statistical criteria (forward selection: ΔOFV > 3.84; backward elimination: ΔOFV > 6.63).

**Figure 2:**
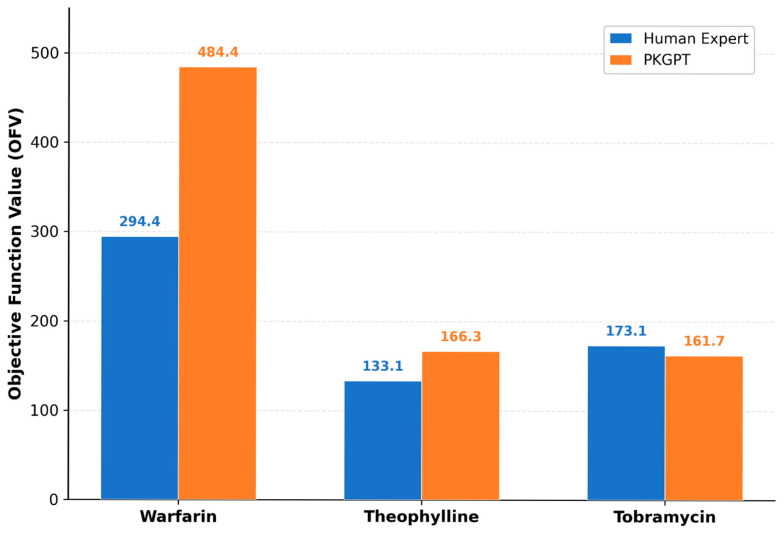
Comparison of objective function values (OFV) between PKGPT and human expert models for Warfarin, Theophylline, and Tobramycin.

The phase transition logic employs specific diagnostic criteria to determine advancement. Within each phase, the orchestrated prompt constrains the LLM to a specialized search neighborhood: Phase 1 focuses on syntax and estimation stability, Phase 2 links residual-pattern heuristics to compartment upgrades, and Phase 4 uses dataset-size-dependent IIV recommendations with shrinkage-based retention thresholds. For example, in the tobramycin Run 1 optimization, PKGPT detected 100% shrinkage on η(Q) and η(V_2_) and removed these unsupported random effects in Phase 3, then simplified the OMEGA structure from correlated to diagonal, yielding the best model (OFV = 161.73). This run completed 9 NONMEM iterations in total; Warfarin Run 1 completed 7 iterations and Theophylline Run 1 completed 6 iterations. Individual PKGPT runs typically complete within 20–40 min (dominated by cumulative NONMEM execution time), compared to days to weeks for expert PopPK model development. Figure 2 illustrates the OFV comparison between PKGPT and human expert models across the three datasets. Complete iteration-stamped control streams and NONMEM output files for all runs are provided in Supplementary File S1.

### 3.3. Warfarin: Numerical Optimization and Parameter Plausibility

The Warfarin dataset (*n* = 32) served as a benchmark case for assessing PKGPT’s ability to handle a standard benchmark oral PK dataset with multiple covariates. In the optimal run, Phase 1 performed data introspection and recommended a one-compartment oral model based on terminal slope analysis, and both PKGPT and the human expert selected the same structural framework (ADVAN2/TRANS2) with FOCE-I estimation. PKGPT adopted a combined (proportional + additive) error model, while the human expert also employed a combined error model with the additive component fixed (σ^2^_add = 0.117 FIX). The human expert model additionally incorporated an absorption lag time (ALAG) and allometric body weight scaling (CL~WT^0.75^, V~WT^1.0^). During iterative refinement, PKGPT initially attempted BLOCK(3) OMEGA with three ETAs, which failed to minimize. The system progressively simplified from correlated to diagonal OMEGA, then reduced to two ETAs with FO estimation, before restructuring with IIV on Ka and CL, fixed V, and reverting to FOCE-I with a proportional-plus-additive error model. PKGPT converged to a final model with OFV 484.43 with successful minimization and covariance step completion. The human expert model achieved a substantially lower OFV of 294.41 (ΔOFV = −189.99), also with successful minimization (157 function evaluations, 4.2 significant digits) and successful covariance step completion.

PKGPT estimated physiologically plausible parameter values (Table 1): Ka = 0.545 h^−1^, CL = 0.133 L/h, V = 7.22 L, with IIV on Ka (ω^2^ = 0.519) and CL (ω^2^ = 0.0711). ETA shrinkage was 42.8% for Ka and 6.9% for CL. The human expert estimated Ka = 1.15 h^−1^ (RSE 30.5%), CL_70_ = 0.135 L/h (RSE 4.8%), V_70_ = 7.86 L (RSE 3.0%), and ALAG = 0.825 h (RSE 8.6%). The expert fixed IIV on Ka (ω^2^ = 0.5 FIX) and estimated IIV on CL (ω^2^ = 0.0643, RSE 37.3%), achieving ETA shrinkage of 32.2% for Ka and 3.3% for CL. The lower OFV was primarily attributable to the allometric body weight covariate and absorption lag time, neither of which PKGPT identified through its automated stepwise procedure (forward selection ΔOFV > 3.84), suggesting limited sensitivity for physiologically established predictors with moderate effect sizes in this sample size.

However, three independent PKGPT runs revealed considerable variability in convergence stability (Supplementary Table S1). While Run 1 achieved OFV 484.43 with a successful covariance step and low shrinkage (6.9% on CL), Runs 2 and 3 produced substantially higher OFVs (1208.94 and 1620.54, respectively) with numerical instability manifesting as collapsed random effects (shrinkage 99.3% and 100%) and failed covariance steps, illustrating the stochasticity inherent in LLM-driven optimization.

### 3.4. Theophylline: Validation with a Small Dataset

The Theophylline dataset (*n* = 12) served as a validation case for sparse, small-sample data. In the optimal run, PKGPT completed optimization and recommended a one-compartment oral model (ADVAN2/TRANS2) with FOCE-I estimation. Phase 1 performed data introspection, estimated λ_z_, and established the base model. Phase 2 confirmed structural adequacy with no evidence of biphasic decline. The initial FOCE-I model with three ETAs and combined error achieved convergence at the first NONMEM execution (OFV = 166.29); subsequent iterations attempted to resolve covariance step failure by simplifying the OMEGA structure to two ETAs and switching to FO estimation, but the original three-ETA model was retained as the best-performing configuration. Phase 4 optimized the IIV structure using a diagonal OMEGA matrix appropriate for the small sample size, and Phase 5 covariate analysis identified no significant covariates given the limited number of subjects.

The final model achieved an OFV 166.29 with a combined error model $(W\;=\;\sqrt{\left(Add^{2\;}+\;{\left(Prop\;\times \;IPRED\right)}^{2}\right)}$) with SIGMA fixed to 1, whereas the human expert employed a simpler proportional error model. PKGPT achieved successful minimization but failed the covariance step (parameter estimate near boundary), while the human expert model achieved a lower OFV of 133.11 (ΔOFV = −33.18) with both successful minimization (148 function evaluations, 3.5 significant digits) and successful covariance step completion. Despite the different error structure, PKGPT parameter estimates were consistent with the expert solution (Table 2): Ka = 1.59 h^−1^ (expert: 1.46, RSE 21.4%), CL = 0.0399 L/h/kg (expert: 0.0404, RSE 8.0%), and V = 1.29 L/kg (expert: 0.465, RSE 4.3%). Both models identified IIV on CL (ω^2^ = 0.0587 vs. 0.0646) and Ka (ω^2^ = 0.409 vs. 0.445). The human expert additionally estimated IIV on V (ω^2^ = 0.0145, RSE 42.2%) with moderate shrinkage (19.4%), while PKGPT’s IIV on V (ω^2^ = 1.06) exhibited extreme shrinkage (85.2%), suggesting overparameterization. ETA shrinkage on CL was 10.0% (PKGPT) vs. 3.8% (expert). Neither model identified significant covariates, consistent with the limited sample size (*n* = 12). The OFV difference primarily reflects stochasticity in error model specification rather than a fundamental difference in structural or pharmacokinetic parameter adequacy.

Three independent PKGPT runs revealed variability in performance (Supplementary Table S2). Run 1 (OFV 166.29) and Run 2 (OFV 179.79) produced consistent CL estimates (both 0.0399 L/h/kg) and Ka estimates (1.59 and 1.49 h^−1^, respectively), demonstrating reproducible point estimation for the primary pharmacokinetic parameters. However, Run 3 yielded a physiologically implausible CL estimate of 2.38 L/h/kg with OFV 898.36 and high shrinkage (70.1%), indicating convergence to a spurious local minimum. Critically, the covariance step failed in all three PKGPT runs (Runs 1–2: parameter near boundary; Run 3: aborted), whereas the human expert model achieved successful covariance step completion. This consistent covariance failure underscores that small datasets (*n* = 12) remain particularly challenging for automated modeling, where limited information results in ill-conditioned Hessian matrices that preclude formal uncertainty quantification.

### 3.5. Tobramycin: Two-Compartment Selection with Parameter Challenges

For Tobramycin, PKGPT correctly identified a two-compartment IV infusion model (ADVAN3) with FOCE-I estimation and a combined (proportional + additive) error model in the optimal run, consistent with the human expert’s structural choice. The human expert model additionally incorporated creatinine clearance (CLCR) and body weight (WT) as covariates ($CL\;=\;TVCL\;\times \;{\left(\frac{CLCR}{58}\right)}^{0.236};V1\;=\;TVV1\;\times \;{\left(\frac{WT}{62}\right)}^{1.07}$). PKGPT achieved OFV 161.73, numerically lower than the human expert model (OFV 173.11, ΔOFV = −11.38); however, this improvement came at the cost of physiological plausibility. Both models achieved successful minimization, but the covariance step failed for PKGPT (boundary) while succeeding for the human expert. The human expert estimated CL = 2.95 L/h (at median CLCR 58 mL/min), V1 = 4.59 L (at median WT 62 kg), Q = 6.85 L/h, and V2 = 13.2 L, all within physiologically plausible ranges (Table 3). PKGPT estimated CL = 2.89 L/h (comparable) but produced a physiologically implausible V2 = 149 L (~10-fold higher than expected) and high V1 = 19.4 L. ETA shrinkage was 30.2% for CL and 70.0% for V1 (PKGPT) vs. 21.2% for CL (expert). PKGPT did not identify CLCR or WT as covariates through its automated stepwise procedure, despite both being established predictors of aminoglycoside pharmacokinetics.

Three independent PKGPT runs showed substantial structural inconsistency (Supplementary Table S3): only Run 1 selected a two-compartment model, while Runs 2 and 3 converged on simpler one-compartment structures (OFV 208.39 and 177.53). Run 2 additionally reverted to first-order (FO) estimation. This structural inconsistency across runs represents a more severe form of stochastic instability than observed in the other two datasets, where repeated runs at least converged on the same compartmental structure. Detailed parameter comparisons and run-to-run stability results are provided in Table 3 and Supplementary Table S3.

## 4. Discussion

The central finding across three case studies is that PKGPT can reliably produce executable, converging NONMEM models, but the gap between numerical optimization and clinically defensible modeling remains substantial. Across three case studies, PKGPT consistently demonstrated that an LLM-driven agent can automate large portions of the NONMEM workflow, including the generation of complete control streams, execution/iteration, and extraction of key diagnostics from .lst outputs. PKGPT demonstrated its capability to achieve numerical optimization in the tobramycin case, where a lower OFV was attained, while in the warfarin and theophylline cases, the human expert achieved lower OFVs through physiologically grounded covariates and parameter constraints.

Regarding the warfarin case, an instructive comparison was observed. Both the human expert and PKGPT selected the same one-compartment oral structure (ADVAN2/TRANS2), yet the human expert model achieved a substantially lower OFV (294.41 vs. 484.43). This difference was attributable to the expert’s incorporation of allometric body weight scaling (CL~WT^0.75^, V~WT^1.0^), an absorption lag time parameter, and strategic variance component estimation. This highlights the distinction between hypothesis-driven and data-driven modeling: human experts leverage *a priori* pharmacological knowledge to incorporate physiologically grounded covariate relationships and constrain the estimation problem, whereas PKGPT relies on automated stepwise procedures. The failure of PKGPT to identify body weight as a significant covariate—despite its well-established role in warfarin pharmacokinetics—suggests that automated stepwise procedures may lack sensitivity for covariates with moderate effect sizes in datasets of limited sample size (*n* = 32). This limitation also reflects a design decision in PKGPT’s covariate procedure, where allometric body weight scaling is evaluated through the same stepwise ΔOFV threshold as all other candidate covariates rather than being incorporated as a default structural feature for body-size-related parameters—an approach that future implementations should reconsider for drug classes with well-established size-based pharmacokinetic relationships.

In the theophylline case, PKGPT produced parameter estimates nearly identical to the expert solution despite selecting a different error model. However, the consistent failure or abortion of the covariance step in the theophylline runs (*n* = 12) underscores a critical distinction between point estimation and statistical precision. While the agent located a plausible minimum for primary pharmacokinetic parameters (Ka, CL) in two of three independent runs, the sparsity of information in small datasets resulted in an ill-conditioned Hessian matrix, preventing the formal calculation of parameter uncertainty, a requirement for regulatory submissions where confidence intervals around key parameters inform dosing recommendations. These patterns illustrate that an automated agent must treat OFV as one signal in a constrained decision process, where plausibility, identifiability, and predictive performance act as hard brakes. Additionally, the volume of distribution was substantially overestimated by PKGPT (V = 1.29 L/kg vs. expert: 0.465 L/kg), accompanied by 85.2% IIV shrinkage on V. Critically, this 85.2% shrinkage level did not trigger Phase 3 activation—which requires ETA shrinkage exceeding 95%—illustrating a fundamental gap between the 20–30% EBE diagnostic reliability threshold established by Savic and Karlsson [30] and the 95% hard trigger currently implemented. This means that substantial shrinkage indicating model misspecification can remain undetected by the automated pipeline. Run 3 further converged at a spurious local minimum with CL = 2.38 L/h/kg—approximately 60-fold higher than Runs 1 and 2—due to poor initial parameterization in Phase 1, an error that the forward-only constraint prevented from being corrected in subsequent phases. The OFV difference between PKGPT and the expert model (ΔOFV = 33.18) was primarily attributable to differences in error model specification rather than structural or pharmacokinetic parameter adequacy, suggesting that automated error model selection represents an additional optimization dimension where explicit decision criteria—analogous to those used for compartment and covariate selection—could improve convergence to clinically preferred model structures.

Similarly, in the tobramycin case, PKGPT achieved a numerically superior OFV of 161.73 compared to the expert model (173.11) by correctly identifying a two-compartment structure. However, this numerical “win” was offset by the estimation of a physiologically implausible peripheral volume (V2 = 149 L), compared to the expert’s 13.2 L. The implausible V2 and Q estimates (149 L and 1.73 L/h, respectively, compared to the expert’s 13.2 L and 6.85 L/h) likely reflect parameter non-identifiability in the peripheral compartment: with limited sampling during the distribution phase, intercompartmental clearance and peripheral volume are poorly constrained, allowing the optimizer to achieve a lower OFV by redistributing variance between Q and V2 without physiological penalty. As an aminoglycoside distributing primarily into extracellular fluid (expected V_2_ ≈ 10–15 L), the 149 L estimate exceeds the expected range approximately 10-fold, exemplifying a “mathematical trap” on a poorly constrained likelihood surface. Phase 3 overfitting detection did not flag this estimate because neither ΔOFV nor shrinkage exceeded their trigger thresholds, indicating the need for a direct physiological plausibility check in the quality scoring system. The concurrent covariance step failure further suggests model over-parameterization with an unstable Hessian matrix. Future implementations should incorporate drug-class-specific parameter bounds and treat covariance step success as a hard constraint. Similarly, the central volume estimate (V1 = 19.4 L) exceeded the expected range for tobramycin in a typical adult (~0.25 L/kg, approximately 15 L at 62 kg), further indicating that the two-compartment parameterization was not adequately constrained. This highlights the potential for automated agents to converge on mathematical minima that lack clinical validity, particularly when the search is not guided by physiologically grounded parameter boundaries. Furthermore, PKGPT’s failure to identify creatinine clearance and body weight as covariates—despite both being well-established predictors of aminoglycoside pharmacokinetics—parallels the pattern observed in warfarin and reinforces the need for domain-informed covariate screening that extends beyond purely statistical stepwise procedures. Both CLCR and WT were explicitly entered into the forward selection procedure (ΔOFV > 3.84, df = 1); neither met the threshold. The structural inconsistency across independent runs, where only one of three runs selected the two-compartment model, represents a more fundamental concern than parameter-level variability, as it indicates that the automated system’s structural decisions are sensitive to stochastic variation in the LLM’s initial model specification.

The stochastic instability observed in repeated runs is another practical limitation. This instability suggests that current LLM agents may occasionally prioritize numerical convergence over statistical robustness. Across all three datasets, run-to-run variability was substantial: Warfarin OFV ranged from 484 to 1621 across three independent runs (with two runs producing collapsed random effects and >99% shrinkage), theophylline produced one run with a physiologically implausible CL estimate 60-fold higher than the expert value, and tobramycin runs did not even converge on the same compartmental structure. This variability may arise from two sources: the inherent stochasticity of LLM text generation (temperature-dependent sampling) and the sensitivity of NONMEM optimization to initial parameter estimates specified in the LLM-generated control stream. Disentangling LLM-level stochasticity from NONMEM-level sensitivity to initial estimates requires controlled experiments with simultaneously fixed LLM temperature, random seed, and NONMEM initialization—a substantially more complex experimental design than the current three-run protocol. Furthermore, temperature = 0 does not guarantee deterministic outputs in API-served LLMs due to floating-point variation across parallel GPU computations. A further fundamental limitation is that LLMs may encode training-corpus biases toward frequently reported model structures, an issue distinct from stochasticity and not addressable through temperature control. From a systems perspective, this argues for consensus strategies such as majority-vote structural selection across multiple runs, median-based parameter initialization from an ensemble of initial runs, or requiring convergence to the same structural model across a minimum number of independent restarts before accepting the final model. The forward-only phase constraint amplifies this variability through a cascade effect: divergent Phase 1 specifications propagate irreversibly through subsequent phases. For example, theophylline Run 3 converged at a spurious local minimum (CL = 2.38 L/h/kg, approximately 60-fold higher than Runs 1–2) due to poor initial parameterization that the forward-only constraint prevented from being corrected.

The performance gap is partly attributable to prompt design: the current covariate prompt relies exclusively on statistical criteria (ΔOFV thresholds), limiting sensitivity for established covariates. Future work should explore pharmacology-informed prompting encompassing three complementary strategies: (1) drug-class-specific covariate checklists (e.g., renal function markers for renally cleared drugs, body size metrics for volume parameters); (2) physiologically plausible parameter bounds informed by drug physicochemical properties; and (3) pharmacological knowledge weighting, where established drug–covariate relationships receive lower statistical thresholds than exploratory covariates. Similarly, structural model components such as absorption lag time should be evaluated as mandatory candidates for oral formulations, as demonstrated by the warfarin case where the absence of ALAG forced Ka to compensate for delayed absorption onset, resulting in higher shrinkage (42.8% vs. 32.2%). Additionally, the modular architecture permits substitution of the underlying LLM; more capable models may further improve covariate identification and reproducibility.

A related limitation concerns the quality scoring system. Adjusting the relative weights among the five existing statistical dimensions (Convergence, Shrinkage, Stability, Precision, Utility) cannot resolve the physiological implausibility problem observed for tobramycin, for a fundamental structural reason: all five dimensions are defined in purely statistical terms, and none evaluate pharmacological plausibility. Concretely, the V_2_ = 149 L estimate for tobramycin did not exceed any of the five statistical triggers (ΔOFV < −50; ETA shrinkage > 95%) regardless of how the weights are redistributed—weight adjustment is therefore an insufficient remedy. When we explored alternative weight configurations during development, increasing Stability or Shrinkage weights penalized some failure modes more effectively but simultaneously degraded performance on datasets where those dimensions were not the primary concern, confirming that the optimal weight configuration is inherently dataset-dependent. Because weights calibrated on a limited number of benchmark datasets cannot be assumed to generalize across the diversity of real-world clinical datasets—which vary substantially in sample size, sampling design, and covariate complexity—systematic validation across broader real-world datasets is planned as a necessary step before recommending any weight recalibration. The root cause is the absence of a pharmacologically grounded criterion in the scoring system itself. To address this in future work, we propose the following concrete development directions: First, addition of a dedicated “Plausibility” scoring dimension that evaluates parameter estimates against drug-class-specific physiological bounds—for example, flagging V_2_ > 30 L as implausible for aminoglycosides, or V/F > 500 L as implausible for oral small-molecule drugs. Second, embedding physiologically informed hard bounds directly as NONMEM $THETA lower and upper limits within the Phase 3 and Phase 4 expert prompts, so that the optimizer is constrained to physiologically plausible parameter spaces before the scoring system is even applied. Third, Bayesian-style informative priors on key PK parameters derived from literature population estimates, which would penalize deviations from established pharmacokinetic ranges during optimization. Systematic calibration of drug-class-specific physiological bounds across multiple drug classes is a prerequisite for deployment of these strategies and represents the highest-priority direction for future development of the PKGPT scoring system. PKGPT differs fundamentally from existing automated PopPK tools in its execution-in-the-loop architecture. Rule-based automation tools such as pyDarwin [28], Pharmpy AMD [25], and SAMBA [27] operate within predefined model search spaces. Specifically, SAMBA accelerates model building by sampling parameter distributions to statistically predict candidate covariates, but it remains constrained to pre-defined compartmental structures and search boundaries. In contrast, PKGPT leverages the generative power of LLMs to dynamically modify and debug NM-TRAN code beyond fixed templates. Neural ODE-based approaches offer data-driven flexibility but function as black-box models that are difficult to interpret and require large training datasets; in contrast, PKGPT produces interpretable NM-TRAN code following established pharmacometric conventions.

Critically, our system implements a closed-loop execution architecture that directly interfaces with NONMEM 7.5, R 4.4 (R Foundation for Statistical Computing, Vienna, Austria), Python 3.12, and PsN 5.3 (Uppsala University, Uppsala, Sweden), enabling automated error detection and iterative code repair. This feature distinguishes PKGPT from prior LLM-based pharmacometrics studies [29,32,33,34,35], which primarily evaluated zero-shot code generation without runtime feedback. However, this autonomous operation introduces the risk of converging to physiologically implausible parameter estimates, where the LLM may propose physically implausible parameter values—as observed in the Tobramycin V2 estimate. This confirms that while the system can control external tools, it still lacks the deep biological intuition required to “vet” its internal diagnostic findings against clinical reality.

A central contribution of PKGPT is that it does not treat NONMEM coding as a single-pass task. The prompt templates encode a “critical checklist” of NONMEM-specific requirements—the kind of domain substrate that generic LLM prompting tends to omit. This directly addresses a known weakness of general-purpose LLM code generation, where performance degrades when the target language is niche or a domain-specific language (DSL).

Several limitations should be acknowledged. First, while PKGPT executes NONMEM locally on the user’s computational environment, it transmits NONMEM control stream code, output summaries (OFV, parameter estimates, shrinkage values), and data-derived summary statistics (*n*, mean, SD per variable) to an external LLM API during each iteration. Raw patient-level data files are not directly uploaded; however, transmitted metadata may contain information derived from clinical data. For environments with strict data privacy requirements (e.g., clinical trial data subject to regulatory restrictions), deployment with a locally hosted open-source LLM would eliminate external data transmission entirely and is identified as an important direction for future implementation. Second, the current implementation supports up to two-compartment models. Real-world applications frequently require more complex structures (e.g., TMDD, transit compartment absorption, indirect response models, time-varying clearance), where the performance gaps observed here would likely be amplified. Extension to such structures would require specialized expert prompts and more sophisticated diagnostic logic. Third, the stochastic instability suggests a need for ensembling. The variability arises from LLM-level stochasticity and NONMEM-level sensitivity to initial estimates, amplified by the forward-only cascade effect. Mitigation strategies include consensus-based structural selection across multiple runs and median-based parameter initialization. The forward-only constraint was motivated by empirical experience during development: unrestricted bidirectional phase transitions caused Phase 2 and Phase 3 to repeatedly trigger each other without convergence, consuming the full iteration budget without producing a final model. A configurable maximum iteration budget (default: 20 iterations per phase) provides an additional termination guarantee. Fourth, PKGPT did not identify certain clinically established covariates. This highlights that even when automated systems identify statistically significant models, the assessment of multicollinearity among candidate predictors and the final judgment on clinical plausibility and biological relevance remain the sole responsibility of human experts. Therefore, human-in-the-loop oversight is not merely a safety feature but a core requirement for final model validation. Based on the failure patterns observed across the three case studies, we propose that the system should flag models for mandatory expert review when any of the following conditions are met: (1) covariance step failure, indicating potential identifiability issues; (2) parameter estimates outside physiologically plausible ranges; (3) structural inconsistency across multiple independent runs; or (4) failure to identify covariates from a mandatory pharmacological checklist. Fifth, the 95% ETA shrinkage threshold used for overfitting detection and phase transition was empirically selected to target structural collapse, but the optimal cutoff within the gap between the 20–30% diagnostic reliability threshold established by Savic and Karlsson [30] and this upper bound remains uncharacterized. Across the three case studies, shrinkage values spanning this intermediate range were observed—42.8% for Ka in warfarin, 85.2% for V in theophylline, and 70.0% for V1 in tobramycin—each carrying different implications for model adequacy that a single fixed threshold cannot fully capture. A systematic sensitivity analysis evaluating model selection quality, false positive and negative rates, and phase transition frequency across a range of shrinkage cutoffs (e.g., 50%, 70%, 80%, 90%) would inform the optimization of this threshold and is warranted as a direction for future research.

## 5. Conclusions

Meaningful automation of NONMEM-based PopPK modeling requires more than single-pass LLM code generation, which typically produces control streams that fail to execute or require extensive manual debugging. PKGPT addresses this gap by embedding pharmacometrics expertise into phase-specific expert-agent prompts and orchestrating them in a phase-structured, forward-only recursive loop that repeatedly executes NONMEM, parses run outputs, and applies targeted repairs. Across three public datasets, this design consistently produced executable models with successful minimization and enabled iterative refinement through automated diagnostics and phase-based model development.

PKGPT demonstrated that an LLM-driven agent can produce executable models with physiologically plausible primary parameter estimates, while benchmarking against human experts revealed persistent gaps in covariate identification, peripheral parameter estimation, and run-to-run reproducibility that necessitate human-in-the-loop oversight for final model acceptance.

Taken together, PKGPT is best viewed as a pharmacometrics copilot that improves executability, speeds early-stage model construction, and reduces the manual burden of iterative debugging and refactoring, while still requiring human oversight for final model acceptance. Future work should prioritize guardrails that align optimization with clinical interpretability, including physiology-informed parameter bounds and priors, mandatory covariate-screening policies for drug classes with well-established drivers, consensus strategies across independent runs to address stochastic variability, stability criteria across restarts, and routine inclusion of predictive diagnostics (e.g., GOF/VPC-based acceptance gates) alongside OFV. With these additions and broader validation across diverse study designs, LLM-based systems could evolve from code generators into reliable modeling assistants that deliver not only executable control streams but also robust, interpretable, and clinically defensible PopPK models.

## Figures and Tables

**Table 1:** Comparison of Pharmacokinetic Parameters for Warfarin: Human Expert vs. PKGPT Agent.

Parameter	Human Expert	PKGPT Agent
Route of Administration	Oral	Oral
Model Structure	1-Compartment + Lag time (ADVAN2)	1-Compartment (ADVAN2)
Estimation Algorithm	FOCE-I	FOCE-I
OFV	294.41	484.43
Ka (h^−1^)	1.15	0.545
CL (L/h)	0.135 (TVCL_70_)	0.133
V (L)	7.86 (TVV_70_)	7.22
ALAG (h)	0.825	—
IIV on Ka (ω^2^)	0.5 (FIX)	0.519
IIV on CL (ω^2^)	0.0643	0.0711
Shrinkage (ηKa)	32.2%	42.8%
Shrinkage (ηCL)	3.3% (Excellent)	6.9% (Excellent)
Error Model	Combined (Prop + Add FIX)	Combined (Prop + Add)
Covariates Identified	WT (allometric)	None

Abbreviations: OFV, objective function value; IIV, inter-individual variability; ω^2^, variance of inter-individual variability; Ka, absorption rate constant; CL, clearance; V, volume of distribution; ALAG, absorption lag time; TVCL_70_, typical value of clearance at 70 kg; TVV_70_, typical value of volume at 70 kg; WT, body weight; FIX, parameter fixed during estimation; FOCE-I, first-order conditional estimation with interaction.

**Table 2:** Comparison of Pharmacokinetic Parameters for Theophylline: Human Expert vs. PKGPT Agent.

Parameter	Human Expert	PKGPT Agent
Route of Administration	Oral	Oral
Model Structure	1-Compartment (ADVAN2/TRANS2)	1-Compartment (ADVAN2/TRANS2)
Estimation Algorithm	FOCE-I	FOCE-I
OFV	133.11	166.29
Ka (h^−1^)	1.46	1.59
CL (L/h/kg)	0.0404	0.0399
V (L/kg)	0.465	1.29
IIV on CL (ω^2^)	0.0646	0.0587
IIV on V (ω^2^)	0.0145	1.06
IIV on Ka (ω^2^)	0.445	0.409
Shrinkage (ηCL)	3.8%	10.0%
Shrinkage (ηV)	19.4%	85.2%
Shrinkage (ηKa)	6.1%	7.5%
Error Model	Proportional	Combined (Prop + Add)
Covariance Step	Successful	Failed (boundary)
Covariates Identified	None	None

Abbreviations: OFV, objective function value; IIV, inter-individual variability; ω^2^, variance of inter-individual variability; Ka, absorption rate constant; CL, clearance; V, volume of distribution; ADVAN2, one-compartment model with first-order absorption subroutine; TRANS2, CL/V parameterization translator; FOCE-I, first-order conditional estimation with interaction.

**Table 3:** Comparison of Pharmacokinetic Parameters for Tobramycin: Human Expert vs. PKGPT Agent.

Parameter	Human Expert	PKGPT Agent
Route of Administration	IV Infusion	IV Infusion
Model Structure	2-Compartment (ADVAN3) CLCR/WT covariate model	2-Compartment (ADVAN3)
Estimation Algorithm	FOCE-I	FOCE-I
OFV	173.11	161.73
CL (L/h)	2.95 ^a^	2.89
V1 (L)	4.59 ^b^	19.4
Q (L/h)	6.85	1.73
V2 (L)	13.2	149
IIV on CL (ω^2^)	0.028	0.0554
IIV on V1 (ω^2^)	—	0.0864
Shrinkage (ηCL)	21.2%	30.2%
Shrinkage (ηV1)	—	70.0%
Error Model	Combined (Prop + Add)	Combined (Prop + Add)
Covariance Step	Successful	Failed (boundary)
Covariates Identified	CLCR, WT	None

^a^ Typical value at median CLCR (58 mL/min); CL = TVCL_med × (CLCR/58)^0.236^. ^b^ Typical value at median WT (62 kg); V1 = TVV1_med × (WT/62)^1.07^. Abbreviations: OFV, objective function value; IIV, inter-individual variability; ω^2^, variance of inter-individual; CL, clearance; V1, central volume; Q, intercompartmental clearance; V2, peripheral volume; CLCR, creatinine clearance; WT, body weight; FOCE-I, first-order conditional estimation with interaction; —, not estimated.

## Data Availability

The data and source code used in this study are openly available in the GitHub repository at https://github.com/Gumgo91/PKGPT, (accessed on 15 April 2026).

## Supplementary Materials

The following supporting information can be downloaded at https://www.mdpi.com/article/10.3390/pharmaceutics18040501/s1. Table S1: Model Stability across Independent PKGPT Runs (Warfarin); Table S2: Model Stability across Independent PKGPT Runs (Theophylline); Table S3: Model Stability across Independent PKGPT Runs (Tobramycin); File S1: PKGPT Iteration Log Files (Warfarin, Theophylline, and Tobramycin).
