# Supplementary material for: PKGPT: Expert-Orchestrated Recursive LLM Agent for Automated NONMEM PopPK Modeling with Human Benchmarking
Source: Pharmaceutics. 2026 Apr 18;18(4):501. doi: 10.3390/pharmaceutics18040501 (PMC13118610; doi:10.3390/pharmaceutics18040501)
Supplement: Supplementary file 1 [file pharmaceutics-18-00501-s001.zip › pharmaceutics-4196965-supplementary.pdf]

Table S1. Model Stability across Independent PKGPT Runs (Warfarin)

**Metric**	**Run 1 (Best)**	**Run 2**	**Run 3**
Structure	1-Compartment	1-Compartment	1-Compartment
Estimation	FOCE-I	FOCE-I	FOCE-I
OFV	484.43	1208.94	1620.54
Ka (h^{-1})	0.545	— (BLOCK)	— (BLOCK)
CL (L/h)	0.133	0.130	0.100
V (L)	7.22	8.00	8.00
IIV on CL (ω^2)	0.0711	—	—
Shrinkage (ηCL)	6.9%	99.3%	100%
Error Model	Combined	Combined	Combined
Covariance Step	Successful	Failed	Failed
Minimization	Successful	Successful	Successful

Abbreviations: OFV, objective function value; IIV, inter-individual variability; ω^2, variance of inter-individual variability (reported as variance, not %CV); Ka, absorption rate constant; CL, clearance; V, volume of distribution; FOCE-I, first-order conditional estimation with interaction.

Table S2. Model Stability across Independent PKGPT Runs (Theophylline)

**Metric**	**Run 1 (Best)**	**Run 2**	**Run 3**
Structure	1-Compartment	1-Compartment	1-Compartment
Estimation	FOCE-I	FOCE-I	FOCE-I

OFV	166.29	179.79	898.36
Ka (h^{-1})	1.59	1.49	1.37
CL (L/h/kg)	0.0399	0.0399	2.38
IIV on CL (ω^2)	0.0587	—	—
Shrinkage (ηCL)	10.0%	3.8%	70.1%
Error Model	Combined	Proportional	Combined
Covariance Step	Failed	Failed	Aborted
Minimization	Successful	Successful	Successful

Abbreviations: OFV, objective function value; IIV, inter-individual variability; ω^2, variance of inter-individual variability (reported as variance, not %CV); Ka, absorption rate constant; CL, clearance; V, volume of distribution; FOCE-I, first-order conditional estimation with interaction.

Table S3. Model Stability across Independent PKGPT Runs (Tobramycin)

**Metric**	**Run 1 (Best)**	**Run 2**	**Run 3**
Structure	2-Compartment	1-Compartment	1-Compartment
Estimation	FOCE-I	FO	FOCE-I
OFV	161.73	208.39	177.53
CL (L/h)	2.89	3.73	3.93
V / V1 (L)	19.4	23.2	23.2
Q (L/h)	1.73	N/A	N/A
V2 (L)	149	N/A	N/A

IIV on CL (ω^2)	0.0554	0.0442	0.0491
IIV on V (ω^2)	0.0864	0.0945	0.0900
Shrinkage (ηCL)	30.2%	—[a]	23.5%
Shrinkage (ηV)	70.0%	—[a]	64.7%
Error Model	Combined	Combined	Proportional
Covariance Step	Failed (boundary)	Failed (boundary)	Successful
Minimization	Successful	Successful	Successful

[a] *ETA shrinkage not reported (FO estimation, covariance step not completed).*

Abbreviations: OFV, objective function value; IIV, inter-individual variability; ω^2, variance of inter-individual variability (reported as variance, not %CV); CL, clearance; V1, central volume; Q, intercompartmental clearance; V2, peripheral volume; CLCR, creatinine clearance; WT, body weight; FOCE-I, first-order conditional estimation with interaction; FO, first-order estimation.
